# Stress Writing Textured Graphite Conducting Wires/Patterns in Insulating Amorphous Carbon Matrix as Interconnects

**DOI:** 10.1038/s41598-017-10294-1

**Published:** 2017-08-29

**Authors:** Ding-Shiang Wang, Shou-Yi Chang, Tai-Sheng Chen, Tung-Huan Chou, Yi-Ching Huang, Jin-Bao Wu, Ming-Sheng Leu, Hong-Jen Lai

**Affiliations:** 1 Material and Chemical Research Laboratories, Industrial Technology Research Institute, Chutung, 31040 Taiwan; 2 Department of Materials Science and Engineering, National Tsing Hua University, Hsinchu, 30013 Taiwan; 3 Metrology Analysis Division, National Nano Device Laboratories, National Applied Research Laboratories, Hsinchu, 30078 Taiwan

## Abstract

This study reports a mechanical stress-based technique that involves scratching or imprinting to write textured graphite conducting wires/patterns in an insulating amorphous carbon matrix for potential use as interconnects in future carbonaceous circuits. With low-energy post-annealing below the temperature that is required for the thermal graphitization of amorphous carbon, the amorphous carbon phase only in the mechanically stressed regions transforms into a well aligned crystalline graphite structure with a low electrical resistivity of 420 μΩ-cm, while the surrounding amorphous carbon matrix remains insulating. Micro-Raman spectra with obvious graphitic peaks and high-resolution transmission electron microscopic observations of clear graphitic lattice verified the localized phase transformation of amorphous carbon into textured graphite exactly in the stressed regions. The stress-induced reconstruction of carbon bonds to generate oriented graphitic nuclei is believed to assist in the pseudo-self-formation of textured graphite during low-temperature post annealing.

## Introduction

New interconnection materials with high electron mobility and a high thermal conductivity are in strong demand for use in miniaturized electronic devices in the post-silicon world. Among many candidate materials for use as next-generation interconnects, crystalline carbonaceous materials, including carbon nanotubes (CNTs), graphene and textured graphite, which exhibit such unique properties as ballistic transportation, a large current carrying capacity (of the order of 10^9^ A/cm^2^), a high thermal conductivity and the ability to be metallic or semiconducting^1–8^, are considered to have the most potential and are sometimes regarded as the “silicon of the future”. In particular, textured graphite has attracted significant attention^4–8^, both theoretically and experimentally, for two main reasons: (1) a lower cost and a simpler process than the the high-temperature and/or complex chemical process for fabricating CNTs and (multilayered) graphene, and (2) outstanding directional/anisotropic electrical and thermal conductivities owing to vast electron delocalization within the carbon layers^9, 10^. The in-plane (along the basal plane) electrical resistivity and thermal conductivity of textured graphite can reach 50 μΩ-cm and ~3000 W/mK, respectively, while the poor cross-plane values are 30,000 μΩ-cm and ~6–50 W/mK^10–12^. Attempts have been made to develop applications that utilize the in-plane rapid transportation of electrons and heat, of textured graphite, such as lithium-ion battery electrodes (vertically aligned graphene layers), interconnects, transistors and heat spreading devices^13–21^.

A potential use of textured graphite is to form interconnections by combining thermal-texturing and patterning processes^19^. In earlier studies, a laser scan-annealing process has been used to thermally induce the localized transformation of amorphous carbon (*a*-C) into graphitic sp^2^ clusters^19^. Thermo-assisted textured graphite patterning processes, such as the heat-induced localized transformation of *a*-C using electron beam deposition and conductive-atomic force microscopy (AFM), have also been proposed in recent years for fabricating interconnects^19–21^. However, some drawbacks that limit the practical application of these techniques, which are yet to be overcome, include (1) a high processing temperature (>700 °C for thermal graphitization of *a*-C), (2) a complex and expensive manufacturing process (difficult to industrialize), and (3) discontinuity of the formed graphitic clusters (a low degree of texturing). Recently, the authors discovered the mechanical stress-induced formation of dispersed, oriented graphitic nanocrystallites in *a*-C films^22^. If graphite can grow from oriented graphitic nuclei, then localized textured graphite may pseudo-self-form only in the mechanical stress-defined regions when low-energy post-annealing is applied (below the temperature of thermal graphitization of *a*-C). Therefore, an opportunity may exist to simply fabricate (direct-write) textured graphite conducting wires/patterns in an insulating *a*-C matrix to form interconnects or even electrodes and heat spreading devices that requires the rapid transportation of electrons and heat.

**Figure 1:**
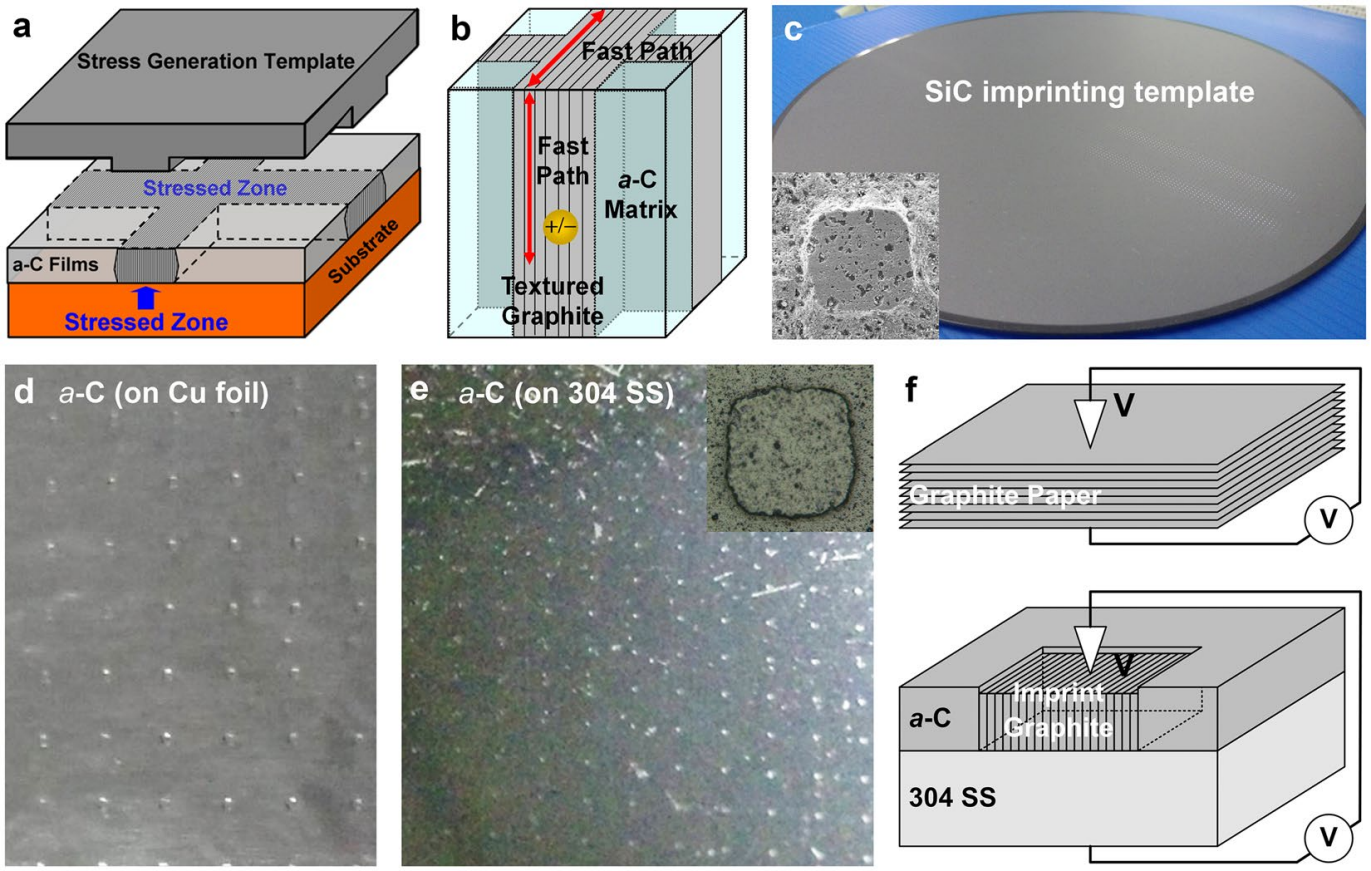
Schematic illustrations of (**a**) imprint-patterning of *a*-C films and (**b**) forming a textured graphite pattern in an *a*-C matrix; (**c**) SiC imprinting template (inset: imprinting bump); SEM images of imprints in *a*-C films on (**d**) Cu foil and (**e**) 304 SS substrates (inset: imprinted dent); (**f**) *I*-*V* characterization (measured vertically through samples).

Therefore in this study, *a*-C films (multilayered, with a Ti underlayer, alternating *a*-C/TiC interlayers and an *a*-C top layer) were deposited on Si, Cu foil and 304 stainless steel (304 SS) substrates using a periodic cathodic vacuum arc system^23^ (see Methods for details of the sample preparation and characterization). A scratch-patterning process, followed by low-temperature post-annealing, was then used to assist in the graphitization of *a*-C exactly in the stressed regions for the direct writing of textured graphite conducting wires (~ 8 μm wide; Supplementary Figure S1, the scratching experiment). The electrical characteristics of the non-stressed regions (denoted as samples TA) and stressed regions (samples SA) of the *a*-C films were measured horizontally along the scratch tracks using a probe station. The bond structure and lattice structure were examined using a micro-Raman spectroscope and a high-resolution transmission electron microscope (TEM), respectively. An imprint-patterning process, followed by low-temperature post-annealing, was utilized to fabricate directly textured graphite conducting patterns (100 μm × 100 μm; Fig. 1, the imprinting experiment). The electrical characteristics were measured vertically through the samples using a conductive-AFM. Based on the experimental results, the opportunity and mechanism of the stress-assisted direct-writing of textured graphite conducting wires/patterns in an insulating *a*-C matrix for potential use as interconnects were studied.

## Results and Discussion

**Figure 2:**
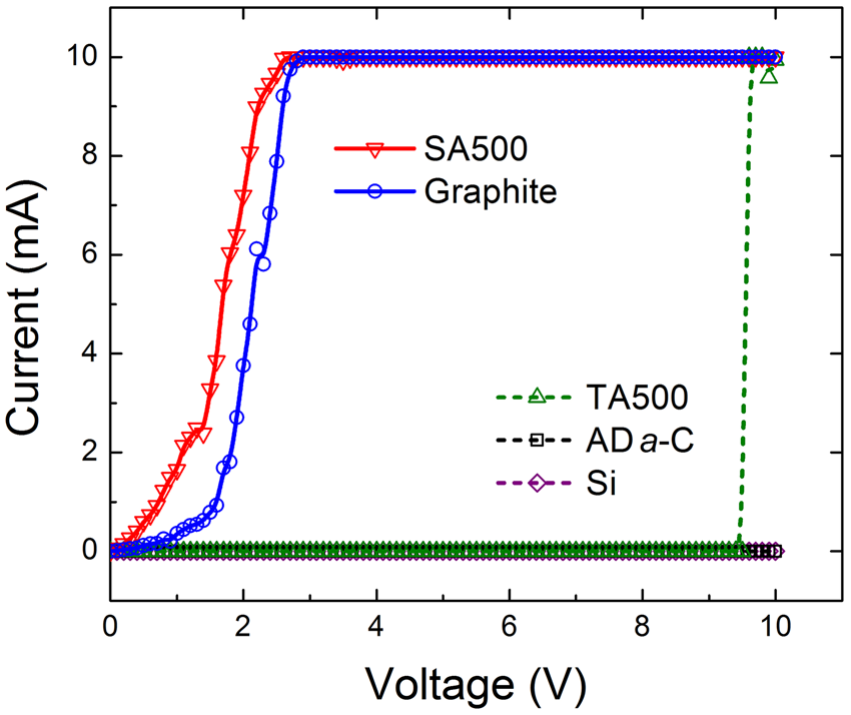
Typical *I*-*V* curves (measured horizontally) of samples of scratching experiment, including Si substrates (purple, insulating), microcrystalline graphite (blue), as-deposited *a*-C films (AD *a*-C, black, insulating), and the non-stressed regions (TA500, green, breakdown at 8–10 V) and stressed regions (SA500, red) of 500 °C post-annealed *a*-C films.

**Figure 3:**
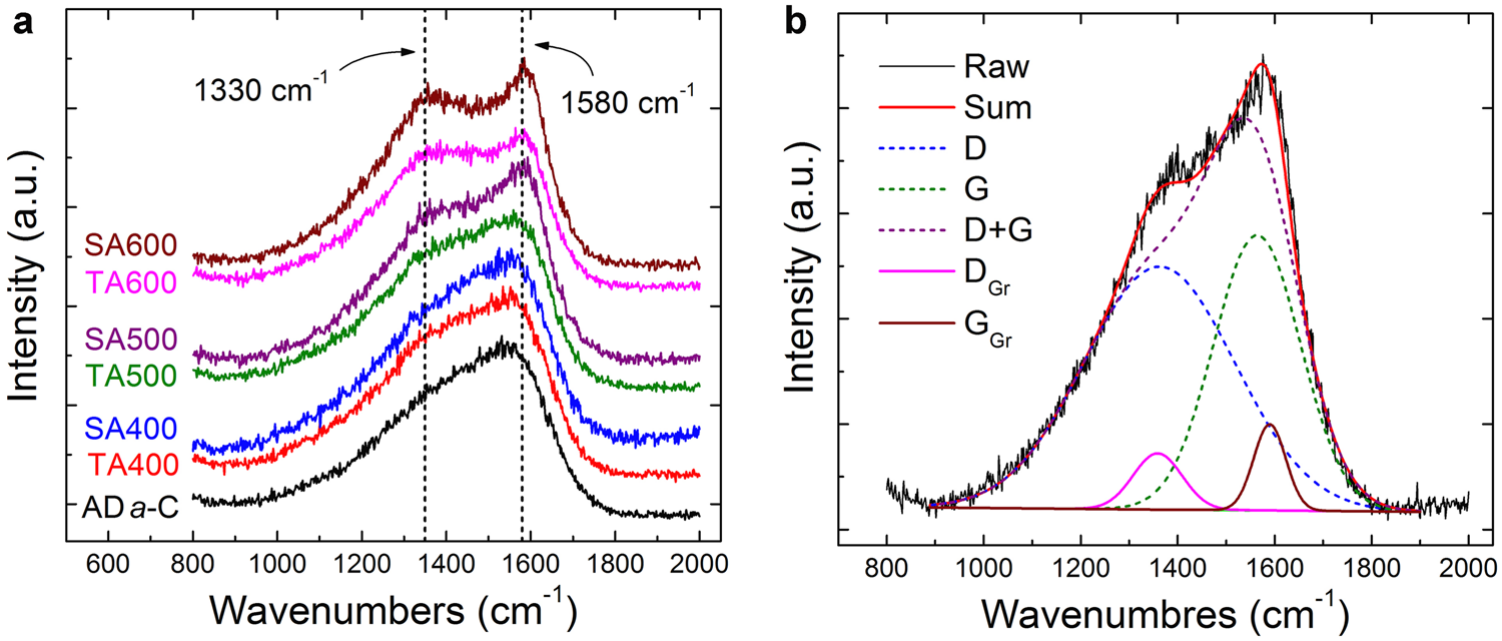
(**a**) Micro-Raman spectra of as-deposited *a*-C films (AD *a*-C) and the non-stressed regions (TA) and stressed regions (SA) of post-annealed *a*-C films (at various temperatures); (**b**) Raman spectrum fittings of the stressed region (SA500) of 500 °C post-annealed *a*-C film (Raw: raw spectrum, Sum: sum of all peak fittings; D and G: broad D and G band fittings, D + G: sum of D and G bands; D_Gr_ and G_Gr_: sharp D and G peak fittings referred to the Raman peaks of microcrystalline graphite).

**Figure 4:**
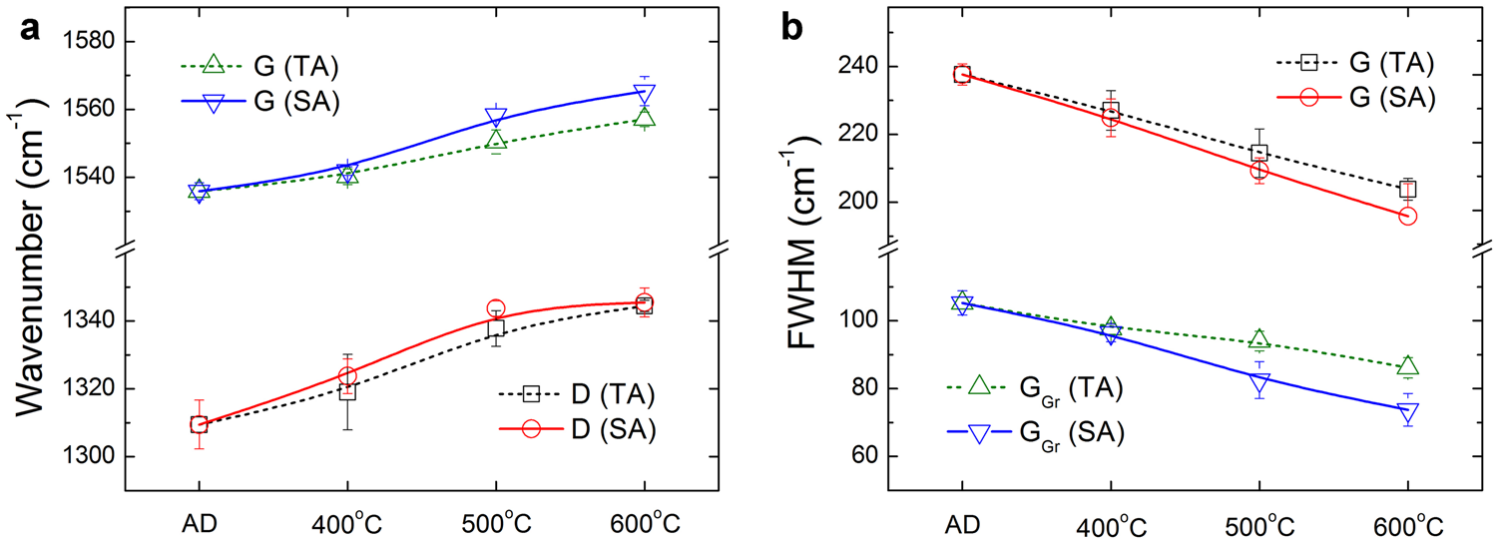
Micro-Raman spectrum analyses of as-deposited *a*-C films (AD *a*-C) and the non-stressed regions (TA) and stressed regions (SA) of post-annealed *a*-C films (at various temperatures): (**a**) wavenumbers of broad D and G bands, (**b**) FWHMs of broad G bands and sharp G_Gr_ peaks.

**Figure 5:**
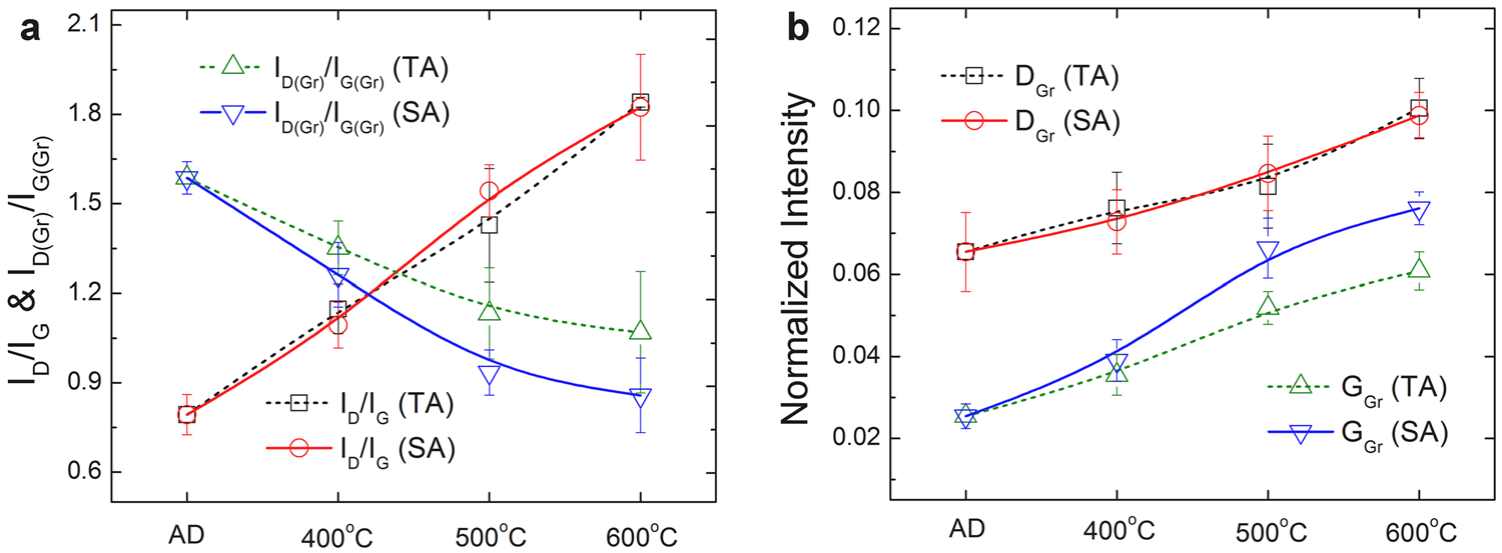
Micro-Raman spectrum analyses of as-deposited *a*-C films (AD *a*-C) and the non-stressed regions (TA) and stressed regions (SA) of post-annealed *a*-C films (at various temperatures): (**a**) D-to-G band intensity ratios (I_D_/I_G_) and D_Gr_-to-G_Gr_ peak intensity ratios (I_D(Gr)_/I_G(Gr)_), (**b**) normalized intensities of sharp D_Gr_ and G_Gr_ peaks (referred to the Raman peak intensities of microcrystalline graphite).

**Figure 6:**
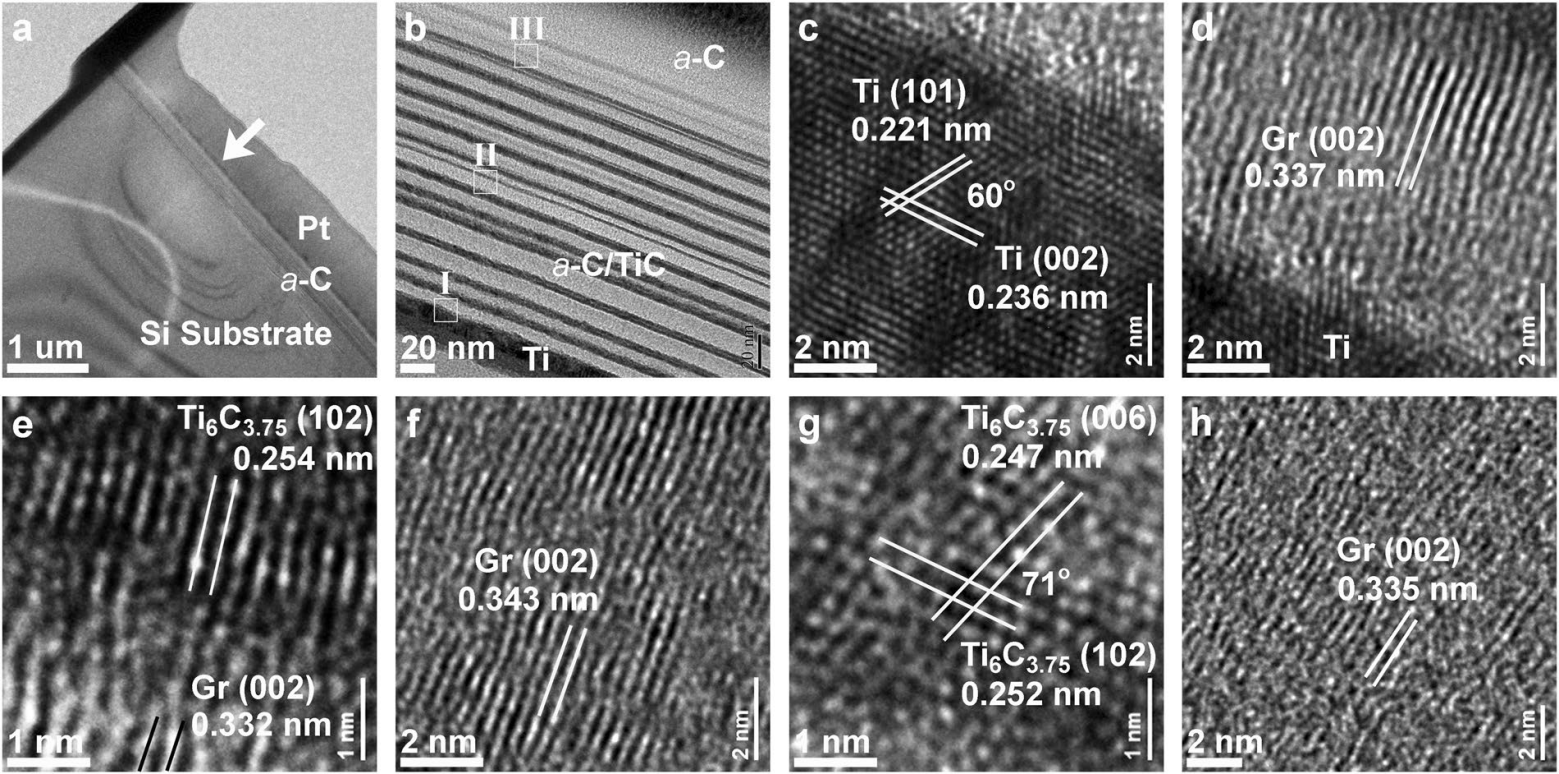
Cross-sectional TEM analyses of the stressed regions (under scratch track, SA500) of 500 °C post-anneal *a*-C films: (**a**) FIB-cut thin foil (arrow: scratch track), (**b**) *a*-C multilayer structure (Ti underlayer, *a*-C/TiC interlayer, *a*-C top layer); lattice images near Ti underlayer (region I): (**c**) Ti, (**d**) *a*-C, (**e**) *a*-C/TiC; lattice images of *a*-C/TiC interlayer (region II): (**f**) *a*-C, (**g**) TiC; (**h**) lattice image of *a*-C top layer (region III; *a*-C transformed into graphite (Gr)).

**Figure 7:**
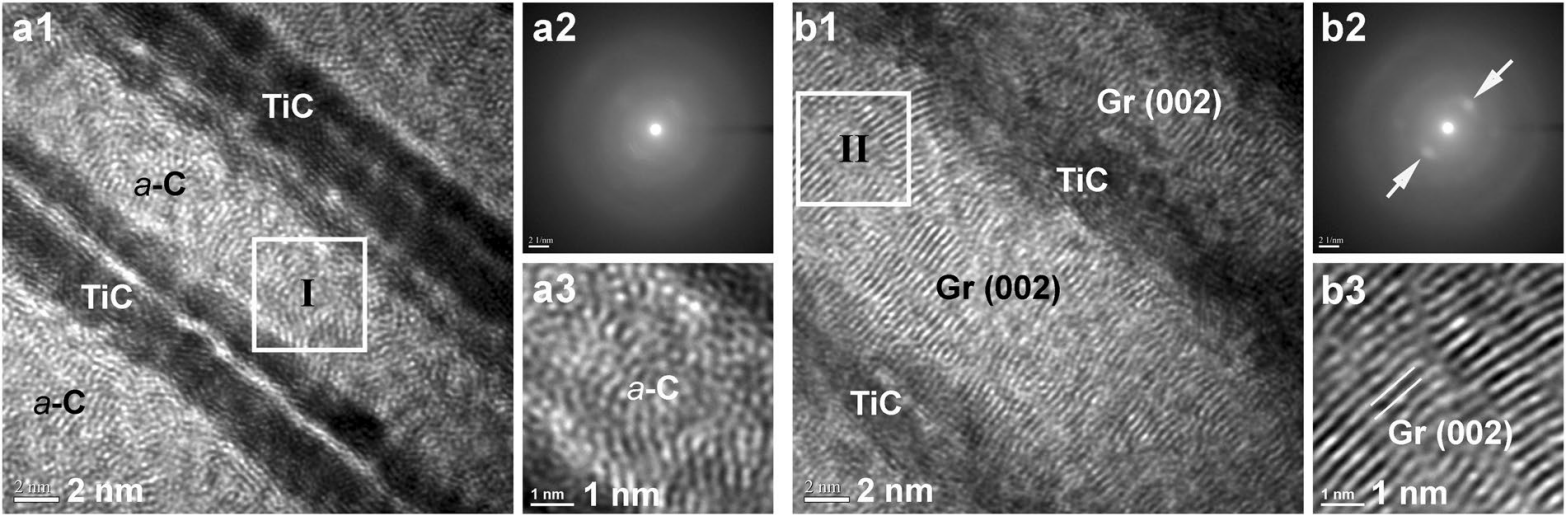
High-resolution TEM analyses of the (**a**) non-stressed regions (away from scratch track, TA500) and (**b**) stressed regions (under track, SA500) of 500 °C post-anneal *a*-C films: (a1,b1) high-resolution images of *a*-C/TiC interlayers; (a2,b2) nano beam diffraction patterns (arrows: diffraction spots of graphite (002) plane); (a3,b3) lattice images of *a*-C interlayer (region I in a1: amorphous structure, region II in b1: textured graphite (Gr)).

**Figure 8:**
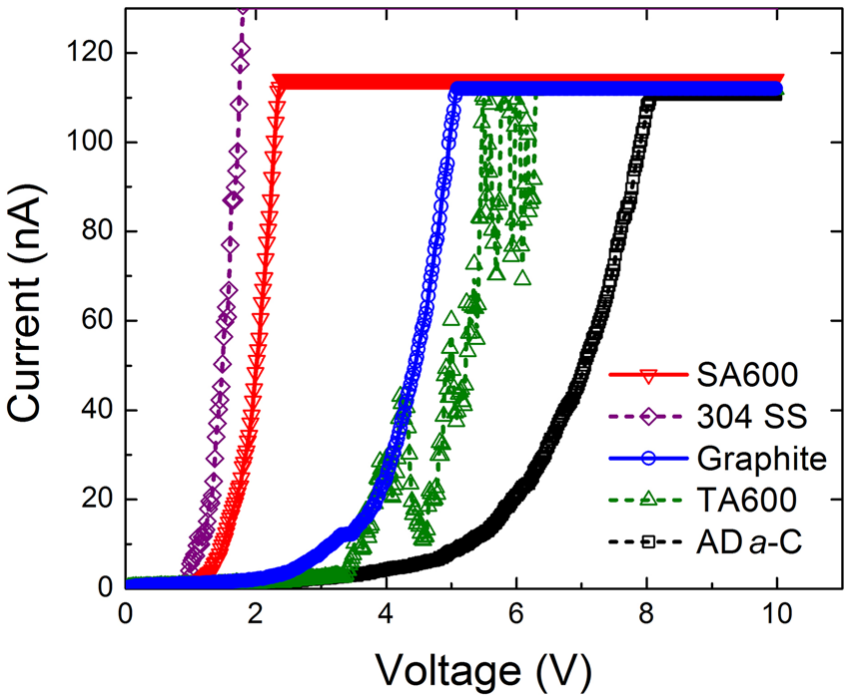
Typical *I*-*V* curves (measured vertically) of samples of imprinting experiment, including 304 SS substrates (purple), graphite paper (blue), as-deposited *a*-C films (AD *a*-C, black), and the non-stressed regions (TA600, green) and stressed regions (SA600, red) of 600 °C post-annealed *a*-C films.

**Figure 9:**
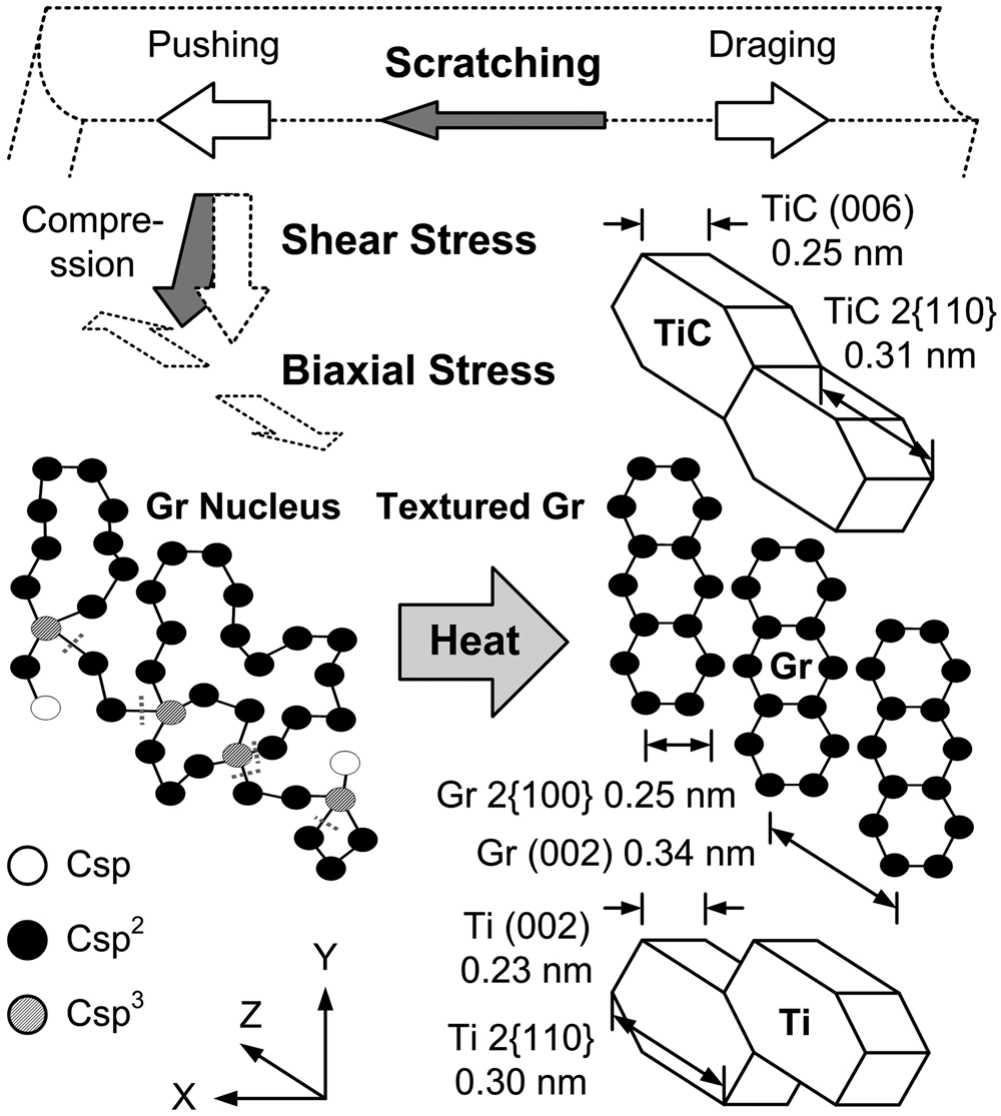
Schematic illustration of stress-assisted transformation of *a*-C into aligned textured graphite during low-energy post-annealing; left: stress-induced formation of oriented graphite nuclei under shear and biaxial stresses; right: textured graphitization with low thermal energy, along with the lattice relationships among graphite (Gr), TiC (Ti_6_C_3.75_) and Ti.

Very interestingly, in the scratching experiment, as indicated by the current-voltage (*I*-*V*) characteristics (Fig. 2, Table 1 and Supplementary Figure S2a), the scratch lines on the 500 °C post-annealed samples (SA500) had an even lower resistivity than the microcrystalline graphite. A graphitic bond structure and an aligned graphitic lattice formed only in the stressed regions, as verified using micro-Raman spectroscopy (Figs 3 to 5 and Supplementary Figures S3) and a high-resolution TEM (Figs 6 and 7). In the imprinting experiment, the electrical characteristics (Fig. 8, Table 2 and Supplementary Figure S2b) also revealed that the imprint patterns on post-annealed samples were more conductive than graphite paper. As more clearly demonstrated below and in Fig. 9 (the stress-assisted textured graphitization mechanism), mechanical stresses assist in the direct-writing (pseudo-self-forming) of textured graphite conducting wires and patterns, embedded in an insulating *a*-C dielectric matrix, for potential use as promising interconnects in future carbonaceous circuits.

**Table 1:** Threshold voltages and electrical resistivities of Si substrate, microcrystalline graphite, as-deposited (AD) *a*-C films and the non-stressed regions (TA500) and stressed regions (SA500) of 500 °C post-annealed *a*-C films (ISL: insulating, BD: breakdown).

Scratching experiment (electrical characteristics measured horizontally on samples)					
Sample	Si substrate	μ-crystalline graphite	AD *a*-C (on Si)	TA500 (on Si)	SA500 (on Si)
Voltage (V)	>10	3.8 ± 1.3	>10	9.2 ± 0.6	2.3 ± 1.1
Resistivity (μΩ-cm)	ISL	477 ± 11	ISL	BD	420 ± 138

Note: threshold voltages: required for ramping up a current to 10 mA; electrical resistivities: acquired at the range of current between 4 and 8 mA.

**Table 2:** Threshold voltages and electrical resistivities of 304 SS substrate, graphite paper, as-deposited (AD) *a*-C films and the non-stressed regions (TA600) and stressed regions (SA600) of 600 °C-post anneal *a*-C films.

Imprinting experiment (electrical characteristics measured vertically through samples)					
Sample	304 SS substrate	Graphite paper	AD *a*-C (on 304 SS)	TA600 (on 304 SS)	SA600 (on 304 SS)
Voltage (V)	1.8 ± 0.2	5.8 ± 1.4	8.1 ± 0.3	6.0 ± 0.1	2.5 ± 0.2
Resistivity (μΩ-cm)	24	2107 ± 666	100 ± 3	87 ± 34	32 ± 4

Note: threshold voltages: required for ramping up a current to 110 nA; electrical resistivities: acquired at the range of current between 40 and 90 nA. The horizontally measured electrical (in-plane) resistivity of graphite paper is 63 ± 3 μΩ-cm.

### Electrical characteristics

Figure 2 and Supplementary Figure S2a plot the *I-V* curves, and Table 1 summarizes the electrical characteristics of the stressed and non-stressed samples in the scratching experiment. As expected, the Si substrate and the as-deposited *a*-C films were insulating. The non-stressed regions of the 500 °C post-annealed *a*-C films (TA500) were also non-conductive and broke down at a high voltage of 8–10 V simply because the post-annealing was conducted below the temperature of thermal graphitization of *a*-C. In comparison, a current was allowed to flow only in commercial microcrystalline graphite and the stressed regions of the ≥500 °C post-annealed *a*-C films (SA500). Obviously, the stressed regions (the scratch lines) were conductive (with a low threshold voltage of about 2.3 V) and had even better electrical characteristics than the randomly oriented microcrystalline graphite. The low resistivity of the stressed regions, 420 μΩ-cm, is of the order of that of in-plane graphite (similar to layered graphene), 250–500 μΩ-cm^8, 24^. Although the resistivity is still higher than the resistivity of state-of-art local Cu interconnects, as recently reported^25, 26^, FeCl_3_ intercalation doping may reduce the resistivity of graphite to 21 μΩ-cm. Prior doping of the *a*-C films followed by use of stress-assisted graphitization may generate doped textured graphite with an even lower electrical resistivity.

### Bond structures

Micro-Raman analyses suggest that the formation of textured graphite to provide a fast transportation pathway of electrons yields the low electrical resistivity of the stressed regions. As is typical, in Fig. 3 and Supplementary Figure S3, the spectra of the as-deposited *a*-C films and the <500 °C post annealed *a*-C films (TA400 and SA400) mainly comprise the broad D and G bands of *a*-C^24^, along with the small graphitic D and G peaks of minor dispersed graphite clusters (defined as D_Gr_ and G_Gr_, with reference to the literature^24, 27, 28^ and the Raman peaks of microcrystalline graphite). In contrast, the “rising-shoulder” spectra of the stressed regions of the ≥500 °C post-annealed *a*-C films (SA500) include an obvious, sharp G_Gr_ peak, which is similar to the thermal graphitization (reordering) of *a*-C films during ≥700 °C annealing (with reconstruction of the sp^2^ and sp^3^ carbon bonds into a stable sp^2^ graphite structure)^21, 27, 28^. Detailed analyses of D and G bands and D_Gr_ and G_Gr_ peaks given in Figs 4 and 5 indicate a distinct difference between the bond structures of the non-stressed regions and the stressed regions of ≥500 °C post-annealed *a*-C films. According to the I_D_/I_G_ ratio and the G peak position of the as-deposited *a*-C films, along with the hardness and modulus (34 GPa and 320 GPa, respectively, measured in the authors’ earlier study^23^), and with reference to the literature^29–31^, the density and sp^3^ fraction are estimated to be approximately 2.4–2.6 g/cm^3^ and 50–60%, respectively, which are close to those of sp^3^-rich *a*-C. The full widths at half maxima (FWHMs) of the G_Gr_ peaks, the D_Gr_-to-G_Gr_ intensity ratios (I_D(Gr)_/I_G(Gr)_) and the normalized G_Gr_ intensities of the stressed samples at ≥500 °C clearly deviate from those of the non-stressed samples, reflecting the fact that mechanical stresses facilitated a substantial change in the bond structure of *a*-C. The slight and gradual variations of the wavenumbers, FWHMs and I_D_/I_G_ intensity ratios of the broad D and G bands with increasing post-annealing temperature revealed minor graphite clustering in the *a*-C matrix that was consistent with the typical reordering of *a*-C by low-temperature annealing^21, 27, 28^. In contrast, the obvious decrease in the FWHM, the significant increase in the intensity of the G_Gr_ peaks, and the drop of I_D(Gr)_/I_G(Gr)_ ratio from 1.6 to only 0.9 (close to the value of microcrystalline graphite, 0.75), all revealed the graphitization of *a*-C only in the stressed regions.

### Lattice structures

High-resolution TEM observations of a clear, aligned graphitic lattice only in the stressed regions of 500 °C post-annealed *a*-C films (SA500), in Figs 6 and 7, confirm the localized phase transformation of *a*-C into textured graphite. In the stressed regions (under a scratch track/dent) of the multilayered *a*-C film (Fig. 6a and b), from the Ti underlayer (Fig. 6c–e), the *a*-C/TiC interlayers (Fig. 6f and g) to the *a*-C top layer (Fig. 6h), three textured structures formed; they were hexagonal Ti (interplanar spacing *d*_(101)_ = 0.221 nm, *d*_(002)_ = 0.236 nm), hexagonal TiC (Ti_6_C_3.75_, *d*_(102)_ = 0.253 nm, *d*_(006)_ = 0.247 nm), and textured graphite (*d*_(002)_ = 0.335–0.343 nm). The textured graphitization of *a*-C in the stressed region was verified by the near-perfect lattice (aligned with the surface, *d*_(002)_ = 0.341 nm) and the obvious nano beam diffraction spots of graphite (002) plane in Fig. 7b. In contrast, a typical disordered structure and diffused halo rings were observed in Fig. 7a, indicating that the amorphous structure remained mostly in the non-stressed region even after the *a*-C films underwent post-annealing at 500 °C.

Notably throughout the stressed regions (from the *a*-C top layer to the *a*-C at the bottom of the *a*-C/TiC interlayers), all of the *a*-C was transformed into a graphitic structure, revealing that the mechanical stresses sufficed to trigger the graphitization of *a*-C even at a depth of several hundreds of nanometers. Also, the aligned graphitization of the *a*-C was found to induce the crystallization of adjacent TiC and Ti in a preferred orientation. Specific lattice relationships among these crystallized structures probably arose from the relaxation of interfacial mismatch stresses, which will be addressed below. Compared to thermo-assisted textured graphitization processes^19–21^ or the pure mechanical stress-induced formation of dispersed graphitic clusters^22^, the presented stress-assisted technique, associated with low-energy post-annealing, generates greater texturing of graphite and is much more promising for fabricating continuous graphite interconnects.

### Imprint-fabrication of conducting patterns

The imprint-patterning process as presented in Methods and Fig. 1a–e, followed by low-temperature post-annealing, is feasible for the fabrication of textured graphite conducting patterns in an insulating *a*-C matrix (deposited on a Cu foil or a 304 SS substrate). The electrical characteristics that were measured vertically through the samples using conductive-AFM (Fig. 1f), shown in Fig. 8, Table 2 and Supplementary Figure S2b, indicate that the imprint patterns on the post-annealed *a*-C films (SA600) are almost as conductive as 304 SS. The patterns even have a higher conductivity and a lower threshold voltage (about 2.5 V, close to the value measured in the above scratching experiment) than commercial graphite paper (out-of-plane). It should be noted that the out-of-plane resistivity of commercial graphite paper is vertically measured to verify the accuracy of measurement using the conductive-AFM. The horizontally measured in-plane electrical resistivity of graphite paper, 63 ± 3 μΩ-cm, is much lower than the out-of-plane resistivity, which is consistent with the literature, which finds that the out-of-plane resistivity of graphite is almost 100 times its in-plane resistivity^32^. Fortunately, the resistivity of sample SA600 along the texture graphite plane is of the same order of magnitude as that of commercial graphite paper along the corresponding plane. In comparison, a much higher threshold voltage is measured in the non-stressed regions of the post-annealed *a*-C films (TA600, ~6.0 V) and the as-deposited *a*-C films (AD *a*-C, ~8.1 V). At 2.5 V, a large current can flow through the imprints, while a very small current leaks in the *a*-C matrix other elsewhere the imprints (TA600 breaks down at about 6 V). The result suggests an opportunity to use the imprints as interconnections, isolated by *a*-C dielectrics, if the input voltage (between 2.5 and 5–6 V, for example) is properly selected during the operation of devices. In addition, the high dielectric constant of the *a*-C matrix that ranges from 4 to 8 (for the present 50–60% sp^3^-rich *a*-C^33–36^) may generate a serious *R*-*C* delay, which must be considered. A feasible means of reducing the dielectric constant to below 2 for potential use in sub-10 nm integrated circuits is to dope *a*-C dielectrics with other elements (such as fluorine)^33–36^. Moreover, the time-dependent dielectric breakdown (TDDB) of the *a*-C films and the maximum current carrying capacity of the stress-formed interconnects affects reliability and requires further investigations.

Importantly, the resistivity (measured vertically) of sample AD *a*-C, TA600 or SA600 combines those of the film and the 304 SS substrate with the contact resistance, so the variation among their resistivities and that of the 304 SS substrate is not marked. However, the resistivity of the imprint patterns is much lower than that of the non-stressed regions. Sample TA600 has a widely fluctuating *I*-*V* curve even at high voltages, which implies that, even if a conductive graphitic phase was formed in the non-stressed *a*-C matrix during 600 °C post-annealing, the graphitic phase was discontinuous. Moreover, the *I*-*V* curves suggest that the samples exhibit different breakdown behaviors. Since the electrical conductivity of *a*-C is governed by the hopping of electrons between dispersive conductive sp^2^ clusters, it depends on the amount and orientation of the sp^2^ bonds^37, 38^. Since continuous textured graphite was formed in the imprint patterns of SA600 but a discontinuous randomly-oriented graphitic phase was formed in sample TA600 (and AD *a*-C), the former is conductive while the latter exhibits soft breakdown with a high threshold voltage of about 6–8 V (and a low current leakage at about 3.5 V). Approximately 1 V is required to trigger current in sample SA600, possibly owing to contact resistance or the activation of electron hopping. The above measurements suggest the high potential of the stress-assisted technique for fabricating textured graphite conduction wires/patterns for use as micro-to-nanoscale interconnects in carbonaceous circuits in microelectronics or even optoelectronics in the future. Appropriate process windows that include wire/pattern dimension, stress scale and post annealing temperature for the fabrication of nanoscale graphitic interconnects with a low electrical resistivity need further investigations.

### Stress-assisted textured graphitization mechanism

An extreme compression is expected to induce the reconstruction of carbon bonds and a phase transformation between graphite and diamond^39^. For the 50–60% sp^3^-rich *a*-C (according to the above Raman analyses) herein, however, the stress-induced reconstruction of carbon bonds (sp^3^ bond breaking and sp^2^ bond formation^31, 40^) is believed firstly to generate oriented graphitic nuclei in *a*-C, which facilitate the growth (pseudo-self-formation) of aligned textured graphite during subsequent low-energy post annealing. The original sp^2^ clusters in the as-deposited sp^3^-rich films are not expected to be the nuclei for the subsequent growth of aligned textured graphite because these clusters are discontinuously dispersed and randomly oriented, and no textured graphitic phase has been identified even in the annealed TA600. The authors’ earlier experiment verified that^22^, at a low sliding speed of 50 μm/sec, the temperature rise that is caused by a small scratching contact is less than 1 °C, and pure mechanical stresses yield dispersed nanocrystallites (clusters) of the graphite phase. As schematically depicted in Fig. 9, the localized scratching^41–45^, or the imprinting (flat punch)^46^, provides not only a normal compressive stress but also a resolved shear stress that depends on the phase angle of the applied load (the scratching possesses a larger shear component, while the imprinting possesses a smaller shear component)^47^. The large resolved shear stress, associated with a large biaxial stress (which involves a residual stress due to the thermal expansion mismatch^8^ and an interfacial stress due to the lattice mismatch among layers^8, 48^), will then cause the reconstruction of carbon bonds and activate the oriented nucleation (clustering) of graphite in *a*-C. The shear stress is likely to cause atom flow and graphitic ring reconstruction along the direction of shear deformation, while the biaxial stress is likely to separate graphite layers, ultimately forming oriented nuclei. An intrinsic compressive stress reportedly favors the formation of sp^3^ bonds during film deposition^49, 50^, however different from the observation herein. The present study concerns a different case in which scratch or imprint stresses are not intrinsic deposition stresses but are externally applied. Previous macroscale friction studies and atomic-scale simulations have all shown that shear stresses can be resolved from scratch or compressive stresses and break sp^3^ bonds to form sp^2^ bonds^51–53^.

After the oriented nuclei are formed, a low thermal energy will then facilitate the aligned growth of the graphite phase^54^ from the nuclei into continuous textured graphite. For elastically anisotropic materials, which include graphite and other hexagonal materials, energy minimization is likely to cause the slidable in-plane graphite layers (in the [100]/[110] directions) to lie parallel to the stresses, whereas the compressible out-of-plane *c* axis (in the [002] direction) lies normal to the stresses^19, 55, 56^. Therefore, in the scratching and imprinting experiments herein, textured graphite layers were formed with an orientation parallel to the compressive stresses. In addition, from the above TEM examinations, the relationships among the lattice orientations of the textured graphite and the recrystallized Ti and TiC are also presented in Fig. 9. To minimize the strain energy that is generated by lattice mismatches, the crystallographic relationships among the graphite, Ti and TiC (Ti_6_C_3.75_) layers herein are (002)_graphite_//2{110}_TiC_//2{110}_Ti_ and 2{110}_graphite_//(006)_TiC_//(002)_Ti_, owing to the similar interplanar spacing of these lattice planes.

The application of pure mechanical stress is a feasible method for transforming *a*-C into discontinuous graphitic nanocrystallites as nuclei; the nuclei (stress orientation dependent) can then assist in the subsequent aligned growth of textured graphite during low-energy post-annealing. However, the ability to apply only stresses to generate directly continuous textured graphite does not exist yet. Nevertheless, future studies that are inspired by this one may develop a more efficient and simpler process for fabricating graphitic interconnects that involve the proper selecting of stress intensity (by increasing it using a smaller/sharper probe) and annealing temperature (by reducing it to the *a*-C deposition temperature). The feasible and practical use of stress-formed conducting textured graphite in sub-10 nm interconnects depends on overcoming the critical challenge of narrowing the wires/patterns. Using very sharp AFM probes to scratch films or fabricating a robust nanoscale mold (by nano-electromechanical means) to imprint films may enable the formation of sub-10 nm interconnects. However, the low speed of AFM scanning, and the great difficulty and low precision/uniformity of imprint mold fabrication may limit the industrial application of this stress-assisted technique, to which end much effort still needs to be made.

### Summary

A mechanical stress-assisted technique for writing textured graphite conducting wires/patterns in an insulating *a*-C matrix was developed with a view to the use of such wires/patterns as interconnects in future carbonaceous circuits. A scratch- or imprint-patterning process with large mechanical stresses was applied to *a*-C films to induce oriented graphitic nucleation and then to assist in the aligned textured graphitization of *a*-C exactly in the stressed regions during low-energy post-annealing below the temperature that is required for the thermal graphitization of *a*-C. The scratch lines and imprints have a textured graphite structure and an electrical resistivity of 420 μΩ-cm, which is lower than that of commercial graphite. Micro-Raman analyses and high-resolution TEM observations confirmed the formation of an aligned graphitic structure in the stressed regions and the retention of an amorphous structure in the non-stressed regions. Also, a stress-assisted textured graphitization mechanism was suggested.

## Methods

*a*-C films (multilayered structure, with a Ti underlayer with a thickness of 27 nm, alternating *a*-C/TiC interlayers with a total thickness of 120 nm and an *a*-C top layer with a thickness of 100 or 500 nm) were deposited on Si, Cu foil and 304 stainless steel (304 SS) substrates using a periodic cathodic vacuum arc system. For the scratching experiment (*a*-C films on an Si substrate, with an *a*-C top layer thickness of 100 nm), as presented in Supplementary Figure S1, a UMIS nanoindenter (Base Model, CSIRO) with a Berkovich diamond tip and a scratch module was used to scratch the multilayered *a*-C films at a constant load of 250 mN and a speed of 50 μm/sec to form wires with a width of about 8 μm wide (with an estimated scratching stress of about 5–6 GPa). For the imprinting experiment (*a*-C films on Cu foil and 304 SS substrates, with an *a*-C top layer thickness of 500 nm), as presented in Fig. 1, an SiC imprint template (5 cm × 5 cm, on an SiC wafer with a diameter of 200 mm) was used to impress the multilayered *a*-C films under a load of 4 tons for 5 s to form patterns of 100 μm × 100 μm (with an estimated imprinting stress of about 13–15 GPa). The scratched/imprinted samples were then post-annealed at 400, 500 or 600 °C at a heating rate of 200 °C/min in a reducing atmosphere of 95% Ar and 5% H_2_ for 30 minutes using a rapid thermal annealing system.

The *I*-*V* characteristics of the non-stressed regions (away from the scratch tracks or the imprints, and denoted as TA samples) and stressed regions (at the scratch tracks or the imprints, and denoted as SA samples) of the *a*-C films that had undergone post annealing were measured, and the electrical resistivities *ρ* were calculated using the equation $\rho =R(A/L)$ where *R* is the resistance, *A* is the cross-section area, and *L* is the length. For comparison, the electrical resistivities of the substrates, as-deposited *a*-C films, microcrystalline graphite and graphite paper were also measured. The electrical characteristics of the samples in the scratching experiment were measured horizontally (along the scratch tracks) using a probe station (Keithley 236; *A*~1.6 × 10^−8^ cm^2^, *L*~40 μm). The electrical resistivities were then acquired from *V*/*I* at currents between 4 and 8 mA. The contact resistance between the probes and the sample surface was estimated, by varying the distance of the two probes, to be nearly zero as compared to the high resistance of the scratch lines with a very large length-to-area ratio *L*/*A*. The electrical characteristics of the samples in the imprinting experiment were measured vertically (through the imprint patterns) using a conductive-AFM (Veeco D3100; *A*~3.9 × 10^−13^ cm^2^, *L*~800.7 μm as the thickness of 304 SS substrates was ~800 μm and the total film thickness was ~0.7 μm). To protect the equipment and probes, a maximum current was set to yield a current density of about 1.25×10^6^ A/cm^2^. Visible micro-Raman spectroscopy (Horiba Jobin-Yvon LabRAM HR; wavelength of 532 nm, spot size of 3–5 μm) was used to detect the bond structures of microcrystalline graphite, the as-deposited *a*-C films and the non-stressed regions and stressed regions of the *a*-C films that had undergone post-annealing at different temperatures. The multiplex peak fitting of the Raman spectra was performed using a Gaussian-Lorentz function. For TEM observations, cross-sectional thin foils were cut from the non-stressed regions and stressed regions (with a top Pt protective layer) of the *a*-C films that had undergone post-annealing at 500 °C using a focused ion beam system (FIB, FEI Nova-200) at an ultralow current of below 30 pA. High-resolution TEM (JEOL JEM-2100F and FEI E.O Tecnai F20 G2) was used to observe their microstructures and lattice structures.

## Electronic supplementary material

Supplementary Information
