# Supplementary material for: Stress Writing Textured Graphite Conducting Wires/Patterns in Insulating Amorphous Carbon Matrix as Interconnects
Source: Sci Rep. 2017 Aug 29;7:9727. doi: 10.1038/s41598-017-10294-1 (PMC5574915; doi:10.1038/s41598-017-10294-1)
Supplement: Supplementary file 1 — Supplementary Information [file 41598_2017_10294_MOESM1_ESM.doc]

**Supplementary Information**

**Stress Writing Textured Graphite Conducting Wires/Patterns in Insulating Amorphous Carbon Matrix as Interconnects**

Ding-Shiang Wang^1^, Shou-Yi Chang^2^,*, Tai-Sheng Chen^1^, Tung-Huan Chou^3^,

Yi-Ching Huang^3^, Jin-Bao Wu^1^, Ming-Sheng Leu^1^, Hong-Jen Lai^1^,*

^1^ Material and Chemical Research Laboratories, Industrial Technology Research Institute, Chutung 31040, Taiwan

^2^ Department of Materials Science and Engineering, National Tsing Hua University, Hsinchu 30013, Taiwan

^3^ Metrology Analysis Division, National Nano Device Laboratories, National Applied Research Laboratories, Hsinchu 30078, Taiwan

* Corresponding authors. Tel.: +886-3-5715131 ext. 33806; fax: +886-3-5722366; e-mail address: changsy@mx.nthu.edu.tw (S.Y.C.). Tel.: +886-3-5916998; fax: +886-3-5820207; e-mail address: hjlai@itri.org.tw (H.J.L.).


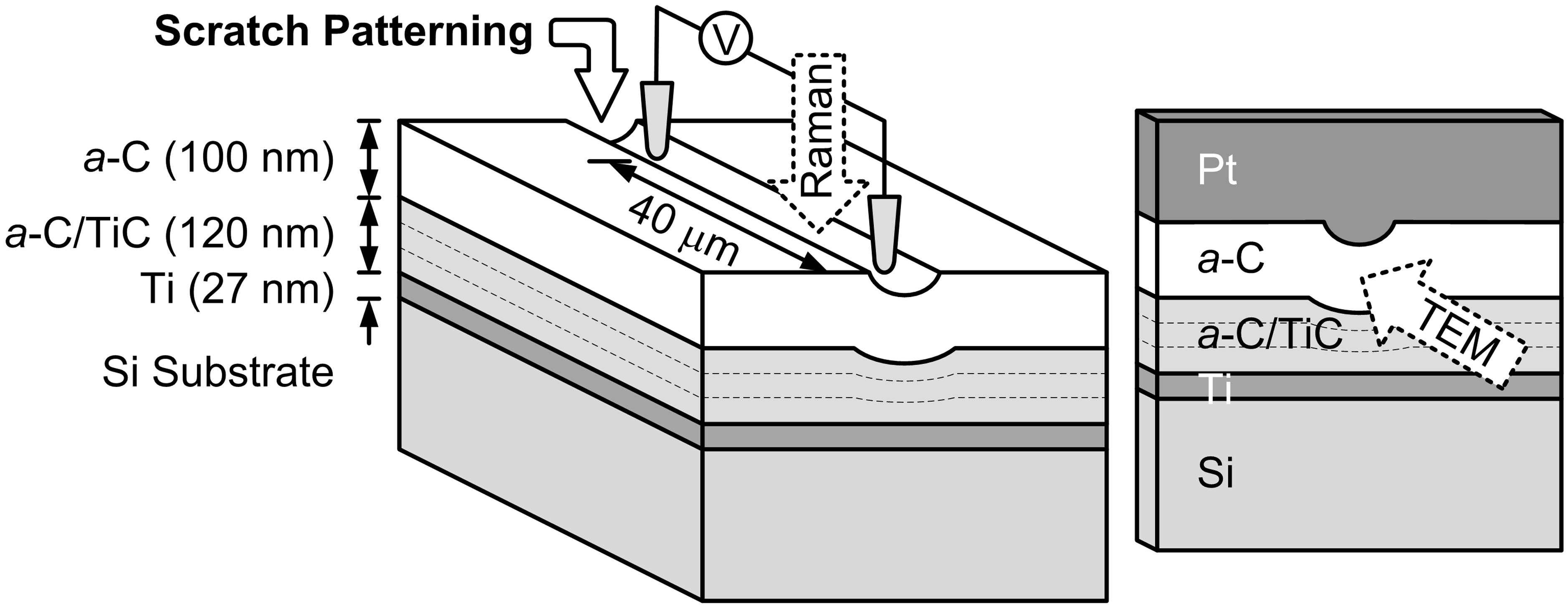


**Figure S1** Schematic illustrations of scratch-patterning of *a*-C films and *I*-*V* characterization (measured horizontally on samples), micro-Raman analyses (focused on scratches) and FIB-cutting of thin foil for cross-sectional TEM observations (under scratches).


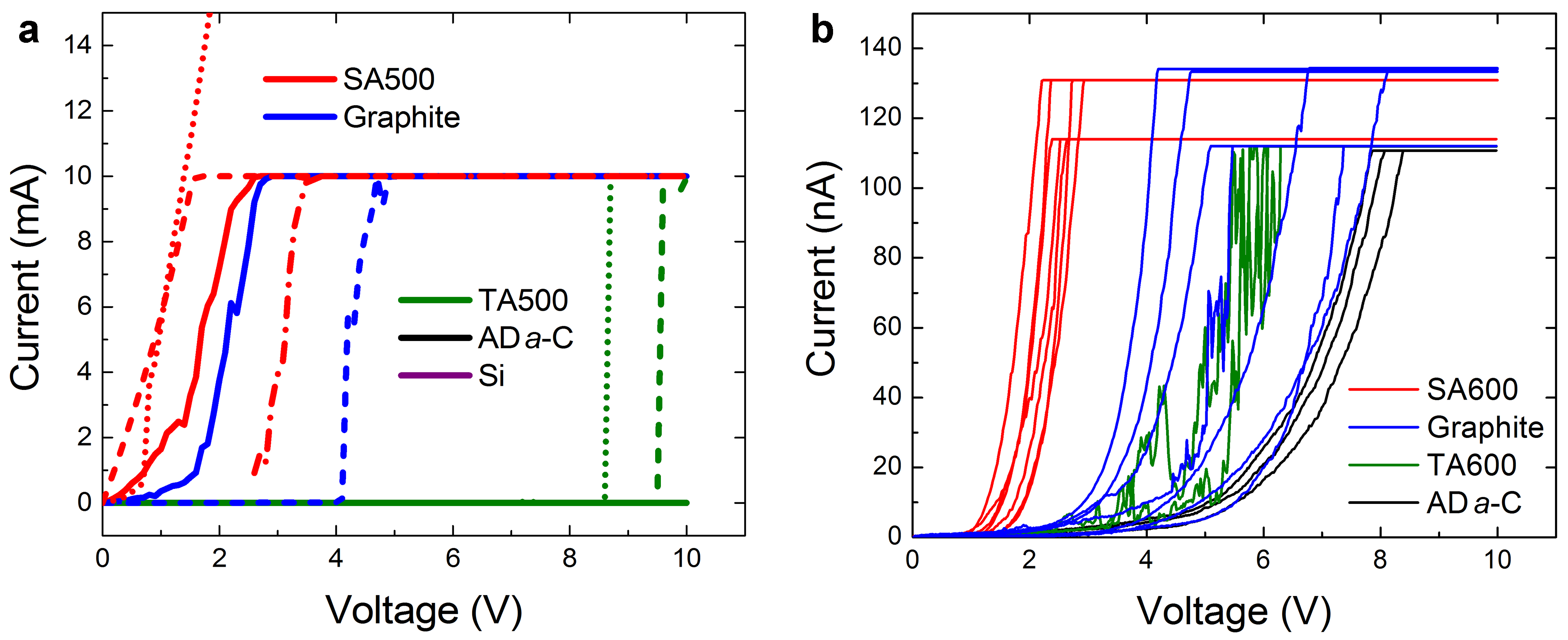


**Figure S2** *I*-*V* curves: (a) for samples of scratching experiment (*I-V* measured horizontally), including Si substrates (purple, insulating), microcrystalline graphite (blue), as-deposited *a*-C films (AD *a*-C, black, insulating), and the non-stressed regions (TA500, green, breakdown at 8-10 V) and stressed regions (SA500, red) of 500°C post-annealed *a*-C films; (b) for samples of imprinting experiment (*I-V* measured vertically), including 304 SS substrates (purple), graphite paper (blue), as-deposited *a*-C films (AD *a*-C, black), and the non-stressed regions (TA600, green) and stressed regions (SA600, red) of 600°C post-annealed *a*-C films.


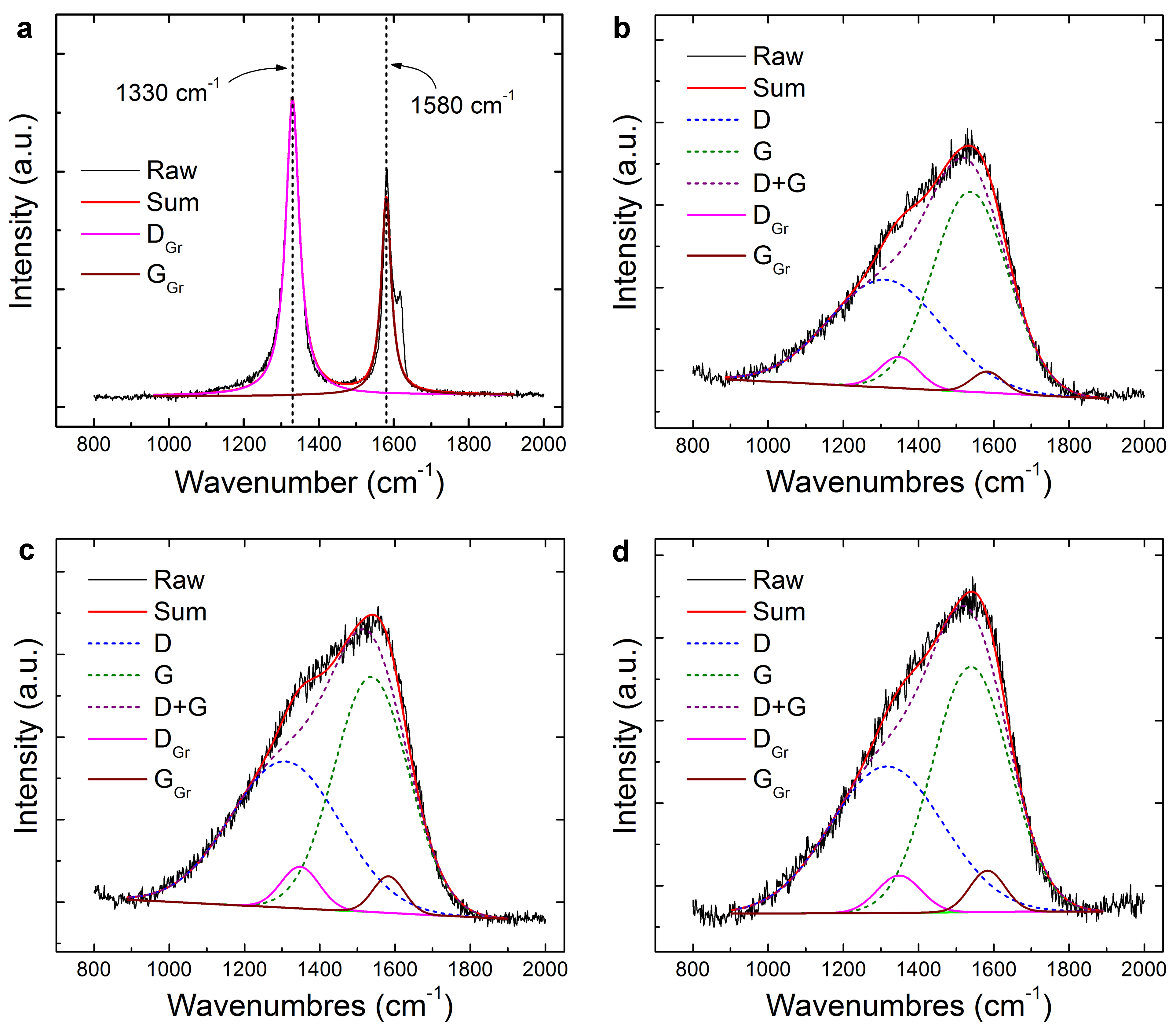


**Figure S3** Micro-Raman spectra and spectrum fittings: (a) microcrystalline graphite, (b) as-deposited *a*-C film; the non-stressed regions (TA) and stressed regions (SA) of post-annealed *a*-C films (at various temperatures): (c) TA400, (d) SA400, (e) TA500, (f) SA500, (g) TA600, (h) SA600 (Raw: raw spectrum, Sum: sum of all peak fittings; D and G: broad D and G band fittings, D+G: sum of D and G bands; D_Gr_ and G_Gr_: sharp D and G peak fittings referred to the Raman peaks of microcrystalline graphite).


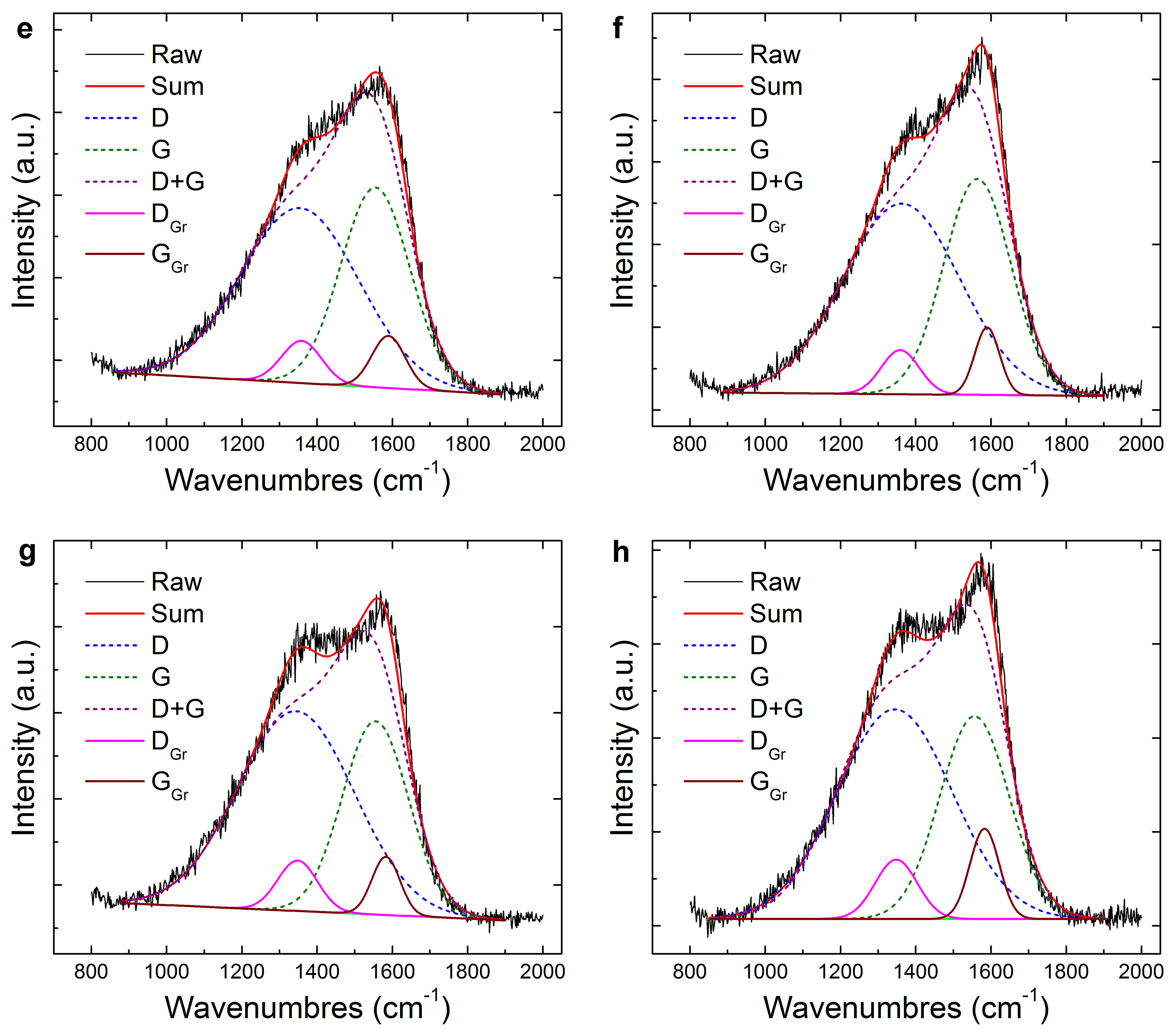


**Figure S3 (continued)** Micro-Raman spectra and spectrum fittings: (a) microcrystalline graphite, (b) as-deposited *a*-C film; the non-stressed regions (TA) and stressed regions (SA) of post-annealed *a*-C films (at various temperatures): (c) TA400, (d) SA400, (e) TA500, (f) SA500, (g) TA600, (h) SA600 (Raw: raw spectrum, Sum: sum of all peak fittings; D and G: broad D and G band fittings, D+G: sum of D and G bands; D_Gr_ and G_Gr_: sharp D and G peak fittings referred to the Raman peaks of microcrystalline graphite).
